# Supplementary material for: Reconciling Mining with the Conservation of Cave Biodiversity: A Quantitative Baseline to Help Establish Conservation Priorities
Source: PLoS One. 2016 Dec 20;11(12):e0168348. doi: 10.1371/journal.pone.0168348 (PMC5173368; doi:10.1371/journal.pone.0168348)
Supplement: S1 Dataset — (ZIP) [file pone.0168348.s002.zip › Taxa/Serra Norte/SN_2013/N4WS/Lista N4WS-57.pdf]

CAVIDADE N4WS-0057

Classe	Ordem	Fam/Outros	Gên/Outros	Espécie	S-U	U-S	Zon.
Arachnida	Acari	(Parasitengonina)		sp.3		X	P
Arachnida	Acari	Argasidae	*Ornithodoros*	sp.1		X	E/P
Arachnida	Amblypygi	Phrynidae	*Heterophrynus*	*longicornis*		2	P
Arachnida	Araneae	Filistatidae		sp.1	X		E
Arachnida	Araneae	Ochyroceratidae	*Ochyrocera*	sp.1	X	X	E/P
Arachnida	Araneae	Pholcidae	*Leptopholcus*	sp.1	X	X	E
Arachnida	Araneae	Pholcidae	*Mesabolivar*	*eberhard*		X	P
Arachnida	Araneae	Salticidae		sp.4	X	X	E
Arachnida	Araneae	Scytodidae	*Scytodes*	*eleonorae*		1	E
Arachnida	Araneae	Symphytognathidae	*Anapistula*	sp.1	X		E
Arachnida	Araneae	Theridiosomatidae		jovem	X	X	E
Arachnida	Opiliones	Escadabiidae		sp.1	X	X	E
Arachnida	Pseudoscorpiones	Chthoniidae	*Pseudochthonius*	sp.1		X	E
Diplopoda	Polydesmida	Pyrgodesmidae		sp.1	X		E
Entognatha	Collembola	Isotomidae		sp.1		X	E
Entognatha	Collembola	Paronellidae		sp.1		X	E
Entognatha	Collembola	Paronellidae		sp.4	X		E
Entognatha	Collembola	Symphypleona		sp.1		X	E
Entognatha	Diplura	Campodeidae		sp.1		X	E
Insecta	Blattodea	Blattellidae		sp.2	4	1	E
Insecta	Diptera	Cecidomyiidae	Cecidomyiinae	sp.	X	X	E/P
Insecta	Diptera	Ceratopogonidae		jovem	X	X	E/P
Insecta	Diptera	Chironomidae		sp.		X	P
Insecta	Diptera	Dolichopodidae		sp.	X		E
Insecta	Diptera	Ephydridae		sp.	X		E
Insecta	Diptera	Mycetophilidae	*Acnemia*	sp.		X	E
Insecta	Diptera	Psychodidae	*Sciopemyia*	*sordellii*	X	X	E/P
Insecta	Heteroptera	Veliidae	*Paravelia*	jovem		X	E
Insecta	Homoptera	Cixiidae		sp.1	X	X	E
Insecta	Hymenoptera	Formicidae	*Anochetus*	sp.1	X		E
Insecta	Hymenoptera	Formicidae	*Hypoponera*	sp.1	X	X	E/P
Insecta	Hymenoptera	Formicidae	*Solenopsis*	sp.2		X	E
Insecta	Hymenoptera	Formicidae	*Strumigenys*	sp.1		X	E
Insecta	Isoptera			sp.	X		E
Insecta	Lepidoptera	Gelechioidea		sp.1		X	E
Insecta	Lepidoptera	Noctuoidea		sp.1		55	E/P
Insecta	Lepidoptera	Noctuoidea		sp.2		X	P
Insecta	Lepidoptera	Tineoidea		sp.3		X	E
Insecta	Lepidoptera	Tineoidea		sp.6	X	X	E/P
Insecta	Orthoptera	Phalangopsidae	*Paraclodes*	sp.		1	E
Insecta	Orthoptera	Phalangopsidae	*Phalangopsis*	sp.		16	E/P
Insecta	Psocoptera	Psocomorpha		jovem		X	E
Insecta	Psocoptera	Psyllipsocidae		jovem	X		E
Gastropoda	Pulmonata	Systrophiidae	*Happia*	sp.	X	X	E
Malacostraca	Isopoda	Armadillidae		sp.1	X		E
Malacostraca	Isopoda	Scleropactidae	*Circoniscus*	*buckupi*		X	P
Symphyla		Scutigerellidae	*Hanseniella*	sp.1		X	E
Amphibia	Anura	Strabomantidae	*Pristimantis*	cf. *fenestratus*	9	6	E
Mammalia	Chiroptera	Emballonuridae	*Peropteryx*	sp.	2	5	E
